# Microencapsulation of a Native Strain of the Entomopathogenic Fungus *Beauveria bassiana* and Bioinsecticide Activity against Pyrethroid-Resistant *Triatoma infestans* to Vector Control of Chagas Disease in the Argentine Gran Chaco Region

**DOI:** 10.3390/tropicalmed8050245

**Published:** 2023-04-24

**Authors:** Linda Vanesa Baldiviezo, Lucía Beatriz Nieva, Nicolás Pedrini, Rubén Marino Cardozo

**Affiliations:** 1 Facultad de Ciencias Naturales, Universidad Nacional de Salta, Av. Bolivia 5150, Salta 4400, Argentina; vanesssabaldi@gmail.com (L.V.B.); rcardozo@salta.gov.ar (R.M.C.); 2 Ministerio de Salud Pública de la Provincia de Salta (MSPS), Salta 4400, Argentina; 3 Instituto de Investigaciones Bioquímicas de La Plata (INIBIOLP), CCT La Plata Consejo Nacional de Investigaciones Científicas y Técnicas (CONICET), Universidad Nacional de La Plata (UNLP), Calles 60 y 120, La Plata 1900, Argentina

**Keywords:** Chagas disease, resistant triatomines, entomopathogenic fungi, microencapsulated, bioinsecticide

## Abstract

The blood-sucking bug *Triatoma infestans* is the main Chagas disease vector in the Southern Cone of Latin America. Populations resistant to pyrethroid insecticides have been detected in the early 2000s and then expanded to the endemic area of northern Salta province, Argentina. In this context, the entomopathogenic fungus *Beauveria bassiana* has been shown to be pathogenic to pyrethroid-resistant *T. infestans*. In this study, both the bioinsecticidal activity and the residual effect of an alginate-based microencapsulation of a native strain of *B. bassiana* (Bb-C001) were tested under semi-field conditions against pyrethroid-resistant *T. infestans* nymphs. Fungal microencapsulated formulation caused higher nymph mortality than the unmicroencapsulated fungus and contributed to maintaining the conidial viability throughout the period evaluated under the tested conditions. These results suggest that alginate microencapsulation is an effective, simple, low-cost method that could be incorporated into the formulation of a bioinsecticide as a strategy to reduce the vector transmission of Chagas disease.

## 1. Introduction

The blood-sucking insect *Triatoma infestans* (Hemiptera: Reduviidae), the main Chagas disease vector in the Southern Cone of Latin America, has been the target of continuous control programs to reduce the risk of disease transmission. The control strategy used has been the indoor application of pyrethroid insecticides [1]. However, it is recognized that this strategy has limited efficacy, mainly in the Gran Chaco area shared by Argentina, Bolivia and Paraguay [2]. Moreover, in the last years, several foci of pyrethroid-resistant *T. infestans* have been documented in wide regions of Bolivia and Argentina [3,4,5]. These difficulties highlight the need to search for new tools to control this vector. The entomopathogenic fungus *Beauveria bassiana* (Ascomycota: Hypocreales) has proven to be useful in field trials, showing successful results after its application in houses infested with pyrethroid-resistant *T. infestans* [6,7], and is therefore a promising alternative for the vector control of Chagas disease in areas with a high degree of resistance to pyrethroids.

The success of a biopesticide of microbial origin lies in a suitable formulation, which depends on the characteristics of the microorganism and the target insect, its relationship with the formulation components, storage conditions and the surface or place of application [8]. In recent years, most research has been focused mainly on the selection of more virulent strains, mass production and field experimentation, leaving aside the search and development of more stable and effective formulations during storage and after application, respectively [9].

In this regard, microencapsulation with biopolymers is among the most innovative tools to improve biological formulations. Microbial microencapsulation is a process by which microorganisms are coated or entrapped within a matrix to protect them from adverse environmental conditions [10,11,12]. Alginate (an anionic polysaccharide extracted from some algae and bacteria) is one of the most natural polymers used in the encapsulation of microorganisms, since it can form gel matrices (in the presence of calcium) around the microorganism [13]. In the case of entomopathogenic fungi, microencapsulation allows them to withstand factors such as solar radiation, temperature and relative humidity that degrade their viability, reducing the dose and number of applications [14,15,16,17].

In this study, the bioinsecticide and residual effect of a microencapsulated formulation based on alginate and conidia of *B. bassiana* were tested on pyrethroid-resistant *T. infestans* under semi-field conditions. This contribution might help substantially improve biological formulations, prolonging their effect and optimizing their effectiveness under field conditions.

## 2. Materials and Methods

### 2.1. Fungus Source and Culture

*Beauveria bassiana* strain Bb-C001 was used in this study. The fungus was isolated from a *T. infestans* cadaver in the Gran Chaco region, Salta province, Argentina (22°16′52.78″ S; 62°42′5.60″ W) [18], and is kept in the Mycological Culture Collection of the School of Natural Sciences, National University of Salta, Argentina.

Aerial conidia were produced by fermentation in solid substrate, using white rice as substrate. Polypropylene bags containing 200 g of rice with 100 mL of distilled water (2:1) were prepared and sterilized in an autoclave at 121 °C at 1 atm of overpressure for 20 min. The bags were inoculated with 20 mL of a conidia suspension with a concentration of 1 × 10^8^ conidia mL^−1^, incubated at 27 °C for a period of 10 to 15 days and dried in an oven at 25 °C for 3 days to reduce humidity to below 5%. Conidia were harvested using a 50-mesh analytical sieve (pore diameter = 297 µm). The concentration per g of material and the initial viability were determined by using a hemocytometer. For viability assay, 5 µL of a conidial suspension was punctually seeded into 15 plates with PDA medium and incubated at 27 °C. Twenty-four hours later, two different areas per plate were observed under a microscope (40×). Germination percentage was calculated as the number of germinated conidia/total number of conidia × 100 [19,20]. Finally, the conidia obtained were packed in 50 mL caramel-colored glass bottles and kept at −4 °C until use. The whole procedure (from sowing to obtaining conidia) was performed in a laminar flow cabinet to avoid contamination.

### 2.2. Fungal Microencapsulation

**Figure 1:**
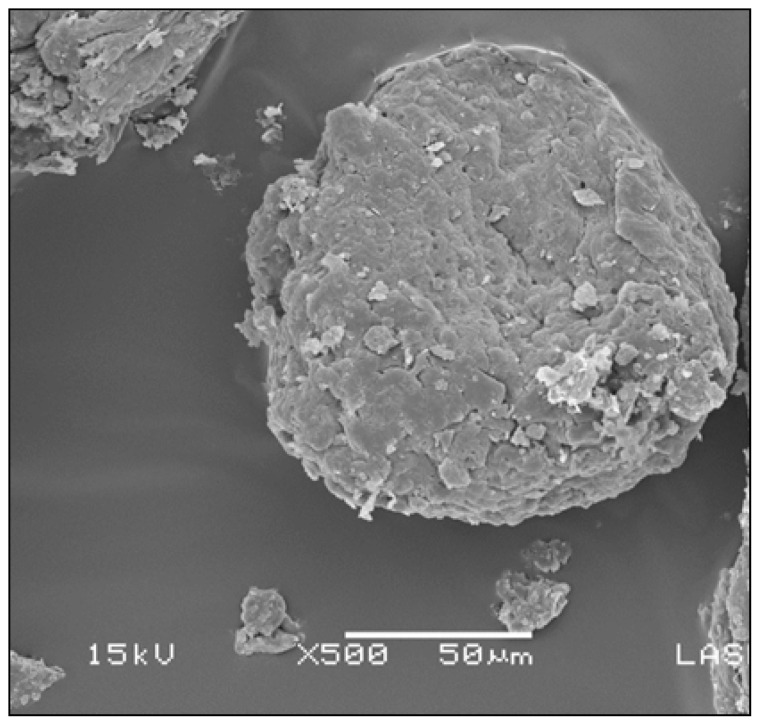
Scanning electron micrograph (SEM) of an alginate microcapsule with *B. bassiana* conidia, scale 50 µm.

Conidial encapsulation was performed by ionic gelation, using sodium alginate as encapsulating matrix and 0.2 M calcium chloride (CaCl_2_), following the procedure described by Carrillo and Bashan with some modifications [21]. One gram of the active ingredient (1 × 10^12^ conidia/g) was mixed with 60 mL of 1% sodium alginate and then stirred up for 30 min. The mixture was sprayed in CaCl_2_ (300 mL) for the formation of microcapsules and allowed to consolidate under constant stirring for 1 h. The microcapsules were filtered and washed three times with sterile distilled water to remove CaCl_2_ residues. They were placed on sterile filter paper in a Petri dish and dried at 35 °C for 72 h. Subsequently, they were observed under scanning electron microscopy (SEM) to observe their structure (Figure 1) and stored in a hermetically sealed container with silica gel until use.

### 2.3. Insects

Fifth instar male nymphs of pyrethroid-resistant *T. infestans* were used. This insect colony comes from insects captured in Salvador Mazza locality, Salta province, Argentina (22°3′0″ S; 63°42′0″ W), and it is maintained in the Insectarium of the School of Natural Sciences, National University of Salta, at 30 °C, 50–60% relative humidity and a photoperiod of 12:12 h (light:dark). The insects were sexed following the procedure described by Brewer et al. [22] and fed with rat blood anesthetized with ketamine. All animal care and experimental laboratory protocols were carried out following the Regulations of the Institutional Committee for the Care and Use of Laboratory Animals and Field Studies (CICUALEC) of the Universidad Nacional de Salta.

### 2.4. Experimental Formulations

Two oil-based fungal formulations of 1 × 10^12^ conidia/g (initial viability of 99%) were prepared: one based on bare conidia (BbC) and the other based on microencapsulated conidia (MicBbC). An oily control without fungus was also prepared. Sunflower oil (Peng and Xia, 2011) was used as an oily vehicle [23], and the polymer Poloxamer 407 (P407) was incorporated into the mixture as a structured vehicle to obtain stable and easily redispersible formulations [24], and finally, diatomaceous earth (DE) was added as a thickener and adjuvant [25]. The proportions of each of the components in formulation are described in Table 1.

### 2.5. Semi-Field Assays

**Figure 2:**
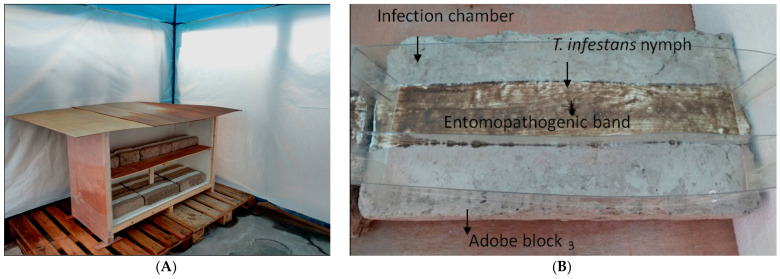
(**A**) Bioassay prototype for triatomine exposure to bioinsecticidal formulations. (**B**) Triatomines were exposed for 1 min on the entomopathogenic band for follow-up post-exposure.

For the semi-field assays, adobe (sun-dried mudbricks) blocks of 8 × 38 × 19 cm (height, length and width, respectively) were used in order to simulate the interior walls of a house built with this material, typical of some native communities living in northern Salta province, Argentina. On the internal face of each adobe block, a surface area of 190 cm^2^ was waterproofed with liquid roofing membrane (SIKA^®^, Buenos Aires, Argentina) and allowed to dry for 24 h. Treatments were randomly assigned in triplicate. The formulations were applied with a brush on the membrane surface, constituting the “entomopathogenic band” (Figure 2B). The treated blocks were placed inside a gazebo for protection from rain, wind and UV radiation, which was located in a sector within the Laboratorio de Investigación y Producción de Biocontroladores (LIPBioc-MSPS, Ministerio de Salud Pública de la provincia de Salta, Argentina) facilities (Figure 2A). The tests were carried out at different times (day 0, 15, 30, 45 and 60) post-application of the entomopathogenic band.

To evaluate the entomopathogenic effect of the formulations, three replicates of 10 insects per treatment were used. The insects were placed on the entomopathogenic band and left in contact for 1 min; this procedure was performed with one insect at a time (Figure 2B). They were then transferred to plastic flasks and incubated at 27 °C, 50–60% RH and a photoperiod of 12:12 h, without feeding. Mortality was registered daily, and the mean mortality percentage and the time for 50% mortality (LT_50_) of insects treated with the formulations were calculated. Dead insects were put in a humid chamber to confirm that death was caused by fungal infection.

For viability and residual effect, samples of the different formulations were taken on the membrane band at the different times tested. Samples of the microencapsulated formulation (MicBbC) were placed in 10 mL of 0.2 M phosphate buffer (pH 7) with constant stirring for complete dissolution of the microcapsules, and the samples of the unmicroencapsulated fungus were placed in 10 mL of a 0.1% Tween 80 solution. Five aliquots (10 µL each) were taken from the suspension of each formulation and seeded in Petri dishes with selective medium (PDA mixed with copper oxychloride, cyproconazole, chloramphenicol and lactic acid to prevent the proliferation of other microorganisms) for triplicate and incubated at 27 °C for 24 h. Germination percentage was determined as described in Section 2.1.

### 2.6. Statistical Analyses

Statistical significance was used for mortality, which was assessed using ANOVA followed by Tukey’s multiple comparison test. LT_50_ was determined by constructing Kaplan and Meier survival curves. To find out if viability was affected by time, the data were arranged in a linear regression model. Graph Pad Prism v.8.0.1 GraphPad Software, San Diego, CA, USA) was used, with a significance level of 0.05 for statistical analyses.

## 3. Results

### 3.1. Entomopathogenic Effect of Both Bare and Microencapsulated Conidia on T. infestans Nymphs

**Figure 3:**
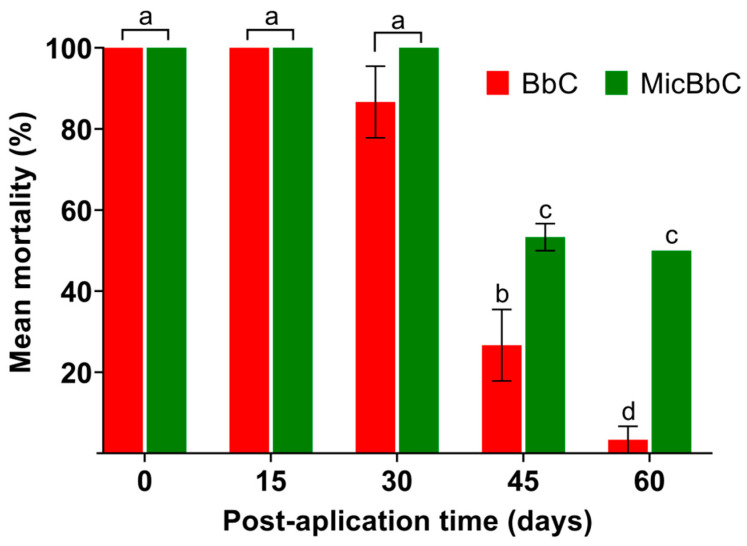
Mean mortality (%) of the *T. infestans* nymphs treated with the *B. bassiana* formulations (BbC and MicBbC) at different post-application time periods. Different lowercase letters indicate significant differences between the different treatments at each time period and of each treatment over time (Tukey, *p* < 0.05). No mortality was observed in controls.

Statistically significant differences in mortality were observed between the evaluated formulations and the different post-application times (*p* < 0.0001). The MicBbC formulation caused, on average, higher nymph mortality than BbC at the end of the bioassays (Tukey, *p* < 0.05). During the first post-application month, high mortality was recorded for both BbC and MicBbC. From the second month onward, the mortality caused by BbC was significantly lower compared to MicBbC, which registered mortalities of about 50% (Tukey, *p* < 0.05) (Figure 3).

In relation to the LT_50_, statistically significant differences were observed according to the Kaplan and Meier tests between the formulations and the post-application times tested (*p* < 0.05). For the BbC formulation in the first month, the LT_50_ ranged between 5 and 10.5 days and were shorter than those observed for MicBbC which varied between 8.5 and 12.5 days. However, in the second month, the LT_50_ could only be calculated for the MicBbC formulation, and it varied between 26 and 28 days (Table 2).

### 3.2. Viability and Residual Effect of Formulations

**Figure 4:**
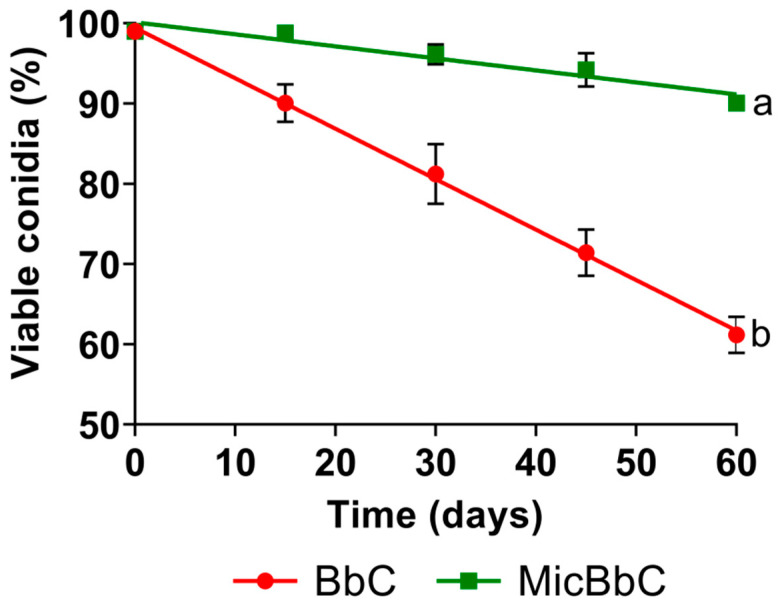
Viability of the experimental formulations (BbC and MicBbC) at different post-application times (0, 15, 30, 45 and 60 days). The negative slope indicates viability loss over time. Different letters indicate significant differences between the slopes of each line (*p* < 0.0001).

Conidia viability (calculated as germination percentage) showed significant differences between the formulations for the different post-application times evaluated (*p* < 0.0001). The differences were observed from day 15 post-application, and the highest viabilities were recorded for the formulated MicBbC, with values that ranged between 90 and 99%, while BbC showed a range between 61 and 90% for the different times tested. Regression analysis showed that there is a significant loss of viability over time that is different for each treatment, MicBbC (b = −0.15% viability day-1; *p* < 0.0001) and BbC (b = −0.63% viability day-1; *p* < 0.0001) (Figure 4).

## 4. Discussion

The effectivity of the chemical control of the Chagas disease vector *T. infestans* based on indoor insecticide spraying as the sole tool is threatened by growing incidents of pyrethroid-resistant population detection [3,4,5]. A successful alternative for *T. infestans* indoor control is the use of the entomopathogenic fungus *B. bassiana*, which has shown good indoor biopesticide performance in field assays and has minimal risk to the environment and other organisms [6,7]. However, the adverse effects potentially caused by environmental conditions to which the fungus may be subjected as a living organism may be an inconvenience for its use in the long term. In this study, we formulated *B. bassiana* conidia in an alginate-based microencapsulation and tested its biopesticide activity against *T. infestans* nymphs on adobe blocks to simulate the interior walls of a typical house of this endemic area.

We found that the microencapsulated formulation (MicBbC) caused, on average, higher mortality in *T. infestans* nymphs than the unmicroencapsulated fungus (BbC) at all post-application times tested. During the first month, no differences were observed between both treatments, which caused high insect mortality. This might be due to that an active initial inoculum, with a high concentration and high viability (1 × 10^12^ conidia/g and 99%, respectively), was used in both treatments. It is well-known that the addition of oil to the fungal formulations as an adjuvant allows one to protect bare conidia, increasing stability and persistence in the conditions tested [23] without affecting their activity or interfering with the infection process [26,27] or the microencapsulation formation process. In the second month, there was an abrupt decrease in mortality in the nymphs treated with the BbC formulated compared to the MicBbC. Bare conidia (BbC formulation) were more exposed to adverse environmental conditions (low humidity and high temperatures), which caused a decrease in conidia survival in the short term and a loss of pathogenicity, in agreement with previous reports in other insects [28,29,30]. Conversely, in the MicBbC formulation, the conidia were protected by the gel matrix that contributed to maintaining their pathogenic capacity for longer periods. These results are also consistent with those reported by other authors [15,31,32].

It was also observed that the LT_50_ were higher for the MicBbC formulation throughout the evaluated period; however, this delay in the development of mycosis due to the slow and gradual release of the encapsulated microorganism did not affect total mortality, in concordance with previous reports [11,17]. On the contrary, the “slow-kill” bioinsecticide effect of the microencapsulated formulation may be advantageous for disease vector control, not only for the obvious extension of the useful life as a pathogen on the action surface but also by reducing the survival of the host without instant killing, which allows the fungus long-term control with lower possibilities of developing resistance [33].

Regarding viability, a greater loss was observed in the BbC compared with the MicBbC formulation for all the times tested. Other studies reported that the encapsulated conidia remain viable during storage, ensuring their persistence over time [16,34,35]. On the other hand, the incorporation of the pharmaceutical excipient P407 as a polymeric vehicle to improve the physical properties of the formulations allowed obtaining stable, homogeneous and redispersible formulations by simple agitation, facilitating its application as a bioinsecticide [36].

## 5. Conclusions

Microencapsulation with alginate proved to be an effective, simple and low-cost method that allows the biological control agent to carry out the infective process on the host more efficiently. These preliminary tests will serve to develop an optimal formulation, which allows maintaining the viability and virulence of *B. bassiana* conidia during storage and after application, which will contribute to a more effective infection of *T. infestans* in the field. Thus, the use of this microencapsulated entomopathogenic fungus in control programs is a promising alternative to reduce the vector transmission of Chagas disease.

## Figures and Tables

**Table 1:** Proportion of components in formulation.

Components	BbC ^1^	MicBbC ^2^	Control
Fungus (conidia/g)	1 × 10^12^	-	-
Microencapsulated (conidia/g)	-	1 × 10^12^	-
Sunflower oil (mL)	30	30	30
P407 (g)	0.59	0.59	0.59
DE (g)	10	10	10

^1^ Fungal unmicroencapsulated formulation; ^2^ Fungal microencapsulated formulation.

**Table 2:** Median lethal time (LT_50_) of the MicBbC and BbC formulations at different post-application time periods.

Time (Days) ^1^	BbC	MicBbC
	LT_50_ (Days)	LT_50_ (Days)
0 ^2^	5	8
15	10	11.5
30	10.5	12.5
45	ND ^3^	26
60	ND ^3^	28

^1^ Time post-application of the formulations on the membrane; ^2^ 24 h after applying the membrane on the adobe blocks; ^3^ ND: not determined because it did not reach 50% mortality.

## Data Availability

Not applicable.
